# Analytical Simulation of the Microbubble Collapsing in a Welding Fusion Pool

**DOI:** 10.3390/ma16010410

**Published:** 2023-01-01

**Authors:** Ahmed Teyeb, Mohamad Salimi, Evelyne El Masri, Wamadeva Balachandran, Tat-Hean Gan

**Affiliations:** 1 Brunel Innovation Centre, Brunel University London, Uxbridge UB8 3PH, UK; 2 Department of Electronic and Electrical Engineering, Brunel University London, Uxbridge UB8 3PH, UK; 3 TWI Ltd., Granta Park, Great Abignton, Cambridge CB 21 6AL, UK

**Keywords:** vibration assisted welding, power ultrasonic, cavitation

## Abstract

This paper explains the use of remote ultrasound vibration at the optimum position and frequencies to vibrate plates under welding, with the aim of initiating cavitation in the molten pool area. It has been shown in the literature that ultrasound cavitation changes microstructure morphology and refines the grain of the weld. In practice, the plates are excited through narrow-band high-power ultrasound transducers (HPUTs). Therefore, a theoretical investigation is carried out to identify the plate-mode shapes due to the ultrasound vibration aligned with the frequency bandwidth of HPUTs available in the marketplace. The effect of exciting the plate at different locations and frequencies is studied to find the optimum position and frequencies to achieve the maximum pressure at the area of the fusion zone. It was shown that applying the excitation from the side of the plate produces an order of $10^{3}$ higher vibration displacement amplitude, compared with excitation from the corner. The forced vibration of cavitation and bursting time are studied to identify vibration amplitude and the time required to generate and implode cavities, hence specifying the vibration-assisted welding time. Thus, the proposed computational platform enables efficient multiparametric analysis of cavitation, initiated by remote ultrasound excitation, in the molten pool under welding.

## 1. Introduction

Most manufacturing processes, such as laser welding, additive manufacturing, and casting, are essentially based on the phenomenon of total fusion of material, followed by rapid solidification. The material’s structure in the final phase and its mechanical and electrical properties strongly depend on the solidification phase [1,2]. This is especially evident when the material consists of an alloy of metals or even a combination of two dissimilar materials, such as in the case of lap joining (by laser welding) of specific connectors to battery cells, for the manufacturing of battery packs for electric vehicles [3]. The difference in melting points between different types of metals leads to the coexistence of liquid and solid phases and subsequently to the agglomeration of particles of similar nature, which ideally should be dispersed homogeneously throughout the liquid phase during solidification [4,5].

The presence of voids and gas, and the grain size variability, are phenomena frequently observed that cause the final material’s properties to deteriorate, leading to problems such as hot cracking and, subsequently, fragile connection [6]. Ultrasonic processing of molten materials is the primary technique that helps to improve the microstructure by grain refinement. The vibration can be transmitted to the molten pool in two ways: vibration of the workpiece [7,8,9] or tool vibration [10,11,12]. Four mechanisms, cavitation, acoustic flow, mechanical effect, and thermal effect, are produced in the fusion zone during ultrasonic vibration. One of the remarkable applications of high-power ultrasound on molten metal is the creation of cavitation in the melt. Acoustic cavitation is a powerful phenomenon promoting melt microheterogeneity and the main processes of degassing and fine filtration in light alloys. The acoustic cavitation in the molten pool contributes to the fragmentation and shaping of intermetallic compounds, which is the leading cause for the degradation of the properties [13]. Four mechanisms are involved in acoustic cavitation: acoustic streaming, microstreaming, microjets, and microstreams.

The associated phenomenon is described as follows. The cavitation bubble expands and contracts in the molten pool until it is exposed to the high-pressure region from the ultrasound wave and then implodes. The implosion of a bubble generates a high-pressure acoustic wave called a shock wave and a local hot spot at a very high temperature. The other mechanisms, such as acoustic streaming, microstreaming, microstreamers, and microjets, are followed by the implosion of the cavitation [14].

The effect of vibration and waves on the creation of a cavitation bubble has been presented in several studies [15,16]. The emission of the shock wave due to the collapse of a cavitation bubble attached to a rigid wall was investigated by Brujan et al. [17]. Their results indicated that a significant portion of the shock wave is dissipated within 100 μm from the bubble wall. Ultrasound cavitation can burst under the compression of the ultrasound wave sooner than they fill with dissolved gas in the melt. Typically, the bubble threshold for collapsing increases as the frequency of ultrasound increases; 1.2 atm at 20 kHz, 1.8 atm at 140 kHz, 3 atm at 1 MHz, and 5.8 atm at 5 MHz [15,16].

Several studies were carried out to characterise the effect of ultrasonic waves on inert tungsten gas (TIG) welding [18,19,20,21]. It was shown by Sun et al. [18] that there is a 300% increase in the penetration rate compared with conventional TIG welding. The increase in the penetration results from increased arc push force via ultrasound, leading to the oscillation in the plasma arc. Ultrasonic-assisted laser welding has been studied by several scholars, for example, [22,23,24,25,26]. Finite element modelling and a laser Doppler vibrometer were used to estimate the ultrasonic energy propagation on the workpiece by Tarasov et al. [26]. An improvement in the microstructure, microhardness, and tensile strength of the 321 stainless steel weld was reported when ultrasonic vibration is transmitted into the welding zone. Lei et al. [25] reported that the weld porosity decreased from 4.3% to 0.9% with a reduction in its average size. Teyeb et al. [27] showed experimentally that weld strength increased by 26% when the laser welding was assisted by ultrasonic vibration. Deeper penetration is reported by Woizeschke et al. [28] and Radel [29] when laser welding is assisted by ultrasonic vibration, which is due to the heating effect of ultrasonic vibration.

The effect of using different ultrasound power on the porosity of a welding joint is experimentally investigated by Yin et al. [30]. The results showed that appropriate ultrasonic energy significantly increases the grain state of the weld. The tensile strength of a welded joint increased by approximately 12% compared with conventional welding. Increasing the ultrasonic power beyond the appropriate level can degrade the tensile strength. As the ultrasound melt treatment before solidification notably affects refining and solidification in light alloys, the welding can be called microcasting.

In this paper, the theoretical vibration of the plate coupled with cavitation vibration in the molten area is presented. As the HPUTs available in the marketplace can excite the plate in a region of narrow band frequency, a simulation study was carried out to understand the wave propagation and modes in the structures at the desired frequencies. An objective of this study is to find the optimum position to remotely vibrate the centre of the plate with the maximum amplitude and hence generate ultrasound capitations in the molten pool area. The cavitation phenomenon that occurs during the solidifying phase is the origin of the microstructure improvements in the joints. Therefore, further theoretical investigations were conducted to find the cavitation vibration at the molten pool area, initiated and vibrated by the remote excitation of the plate. Such a theoretical investigation can help to find the position, frequency, and time for vibration-assisted welding.

## 2. Theoretical Modelling

**Figure 1:**
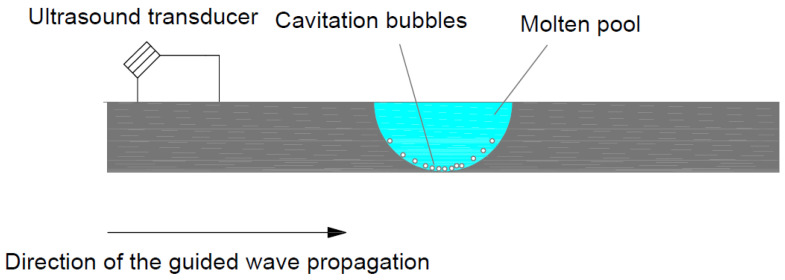
Leakage of the guided wave into the fluid. The water boils due to low local pressure, which generates cavitation.

This section provides theoretical explanations of the plate’s vibration under ultrasonic-guided wave propagation and its interactions with the ultrasound cavitation in the fusion zone. As shown in Figure 1, part of the ultrasound-guided wave travelling in the plate leaks or refracts into the liquid. When the sound pressure of the UGW (ultrasonic-guided waves) exceeds the cavitation threshold, several microbubbles are generated in the fluid close to the solid–fluid boundary. As the ultrasound excites the plate, the microbubbles are subjected to positive and negative pressure. They expand and contract under negative and positive pressure, respectively [31,32].

### 2.1. Ultrasonic Plate Vibration

Modelling of plate vibration has been studied extensively since 1787 [33]. Most of the relevant background theory on the free and forced plate vibration can be found in the reference work by Leisaa [34]. Owing to the presence of the higher order matrices, the virtual approach is suggested by Sung et al. [35,36,37] in a series of studies, making it computationally less expensive to estimate the response of the plate. In this study, the theoretical modelling from Vlasov [38] is used to obtain the mode shapes of the plate at the desired frequency region. Based on the principle of virtual work, the steady-state transverse displacement, ξ(x,y) of a full clamped plate subjected to harmonic point excitation at $\xi \left(x^{\prime },y^{\prime }\right)$ is:

(1) $$\xi \left(x,y\right)=F_{0}\overset{\infty }{\underset{m=1}{\sum }}\sum_{n=1}^{\infty }\frac{\Psi_{mn}\left(x,y\right)\Psi_{mn}\left(x^{\prime },y^{\prime }\right)}{B(I_{1}I_{2}+2I_{3}I_{4}+I_{5}I_{2}-\rho_{s}\omega^{2}I_{2}I_{6})}$$

where $F_{0}$ is the force amplitude, *B* is the bending stiffness $B={\mathrm{Eh}}^{3}/12\left(1-v^{2}\right)$, E is Young’s modulus, v is the Poisson ratio, $\rho_{s}$ is the plate density and ω is the angular frequency.

The eigenfunctions can express the shape function associated with the plate:

(2) $$\Psi_{mn}\left(x,y\right)=\vartheta_{m}\left(x\right)\zeta_{n}\left(y\right).$$

The $\vartheta_{m}$ and $\zeta_{n}$ parameters can be expressed by Bessel and Hankel functions, respectively:

(3) ϑm(x)=J(Bmxa)−J(βm)H(βm)Hβmxa,,ζn(y)=J(βnyb)−J(βm)H(βm)H(βnyb),

where: J(s)=cosh(s)−cos(s) and H(s)=sinh(s)−sin(s), and $\beta_{n}$ is the n-th root of cosh(β)cos(β) = 0. The I parameter in the denominator of Equation (1) can be expressed by:

(4) $$I_{1}=\overset{a}{\underset{0}{\int }}\vartheta_{m}^{\prime \prime \prime }\vartheta_{m}dx,\;I_{2}=\overset{b}{\underset{0}{\int }}\zeta_{n}^{2}\;dy,\;I_{3}=\overset{a}{\underset{0}{\int }}\vartheta_{m}^{\prime \prime }\vartheta_{m}dx,\;I_{4}=\overset{a}{\underset{0}{\int }}\zeta_{n}^{\prime \prime }\zeta_{n}dx,\;I_{5}\;=\overset{b}{\underset{0}{\int }}\zeta_{n}^{\prime \prime \prime }\zeta_{n}dy\;I_{6}=\overset{a}{\underset{0}{\int }}\vartheta_{m}^{2}dx.$$

The natural frequency of the plate can be estimated using the eigenfunctions:

(5) $$\omega_{mn}=\sqrt{\frac{B}{\rho_{s}}}\sqrt{\frac{I_{1}I_{2}+2I_{3}I_{4}+I_{5}I_{6}}{I_{2}I_{6}}}.$$

A high-power ultrasound transducer (HPUT) can generate ultrasonic compressional vibration at a narrow band frequency. It is advantageous to select the transducer to align with the resonance frequency of the plate that can generate the high-pressure acoustic wave in the fusion zone of the weld.

**Figure 2:**
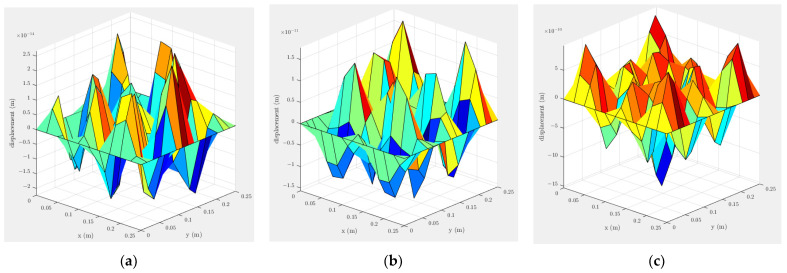
A clamped-plate vibration-mode shape subjected to unit force excitation at the (**a**) corner, (**b**) side and (**c**) centre of the plate at 20 kHz. The excitation locations are illustrated in Figure 3.

**Figure 3:**
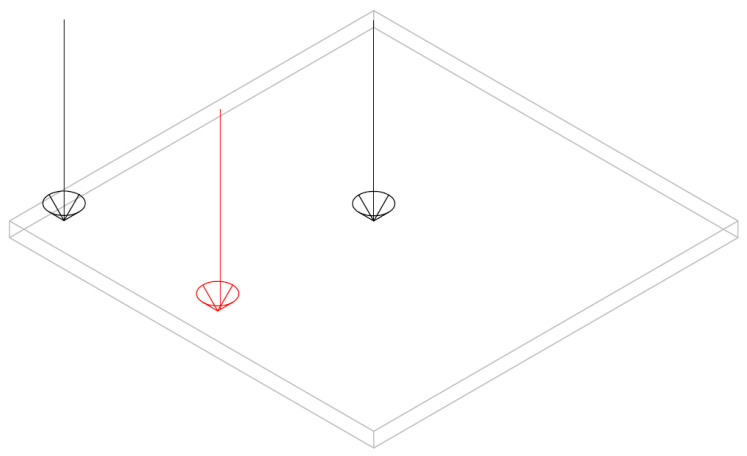
Clamped aluminium alloy plate subjected to ultrasonic excitation at three locations.

Figure 2 shows a 25 cm×25 cm×0.1 cm clamped aluminium alloy plate-mode shape with alloy density $\rho_{s}$ = 2800 kg/m^3^, Poisson ratio ν = 0.33 and Young modulus E = 72.5 GPa, subjected to unit force at the locations illustrated in Figure 3. Although in ultrasonic melting treatment different frequency bandwidth are employed to excite the plate, in this study the plate-mode shapes are plotted for the frequency 20 kHz, subjected to a unit force at the locations illustrated in Figure 3.

As illustrated in Figure 2, at the excitation point the displacement reaches its maximum value. Although using a vibration close to the tip of the laser head is preferable, it might not be practical to be very close to the laser head due to the heating produced in the welding zone area. Another approach is to use a fixed location for the ultrasound to vibrate the fusion zone area remotely. As illustrated in Figure 2b,c when the plate is subjected to excitation from the side, the displacement amplitude is an order of $10^{3}$ higher compared to the corner excitation.

**Figure 4:**
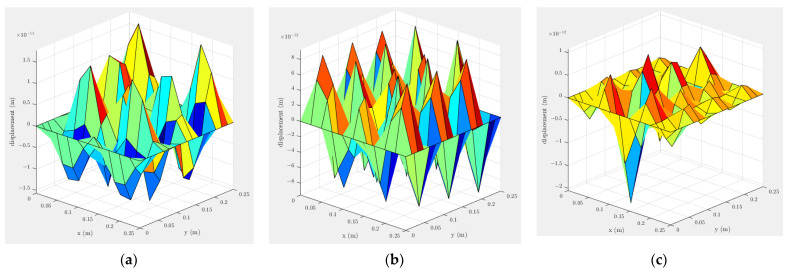
Excitation from the side using a single transducer at (**a**) 20, (**b**) 28 and (**c**) 40 kHz. The excitation location is highlighted by a red arrow in Figure 3.

Ahmed et al. [27] used an ultrasound transducer at the side of a plate to transmit the ultrasound vibration into the molten pool. In this study, the effect of a single transducer on the plate displacement is plotted at the frequencies used by Teyeb et al. [27]: 19, 28 and 40 kHz. The excitation location is highlighted by a red arrow in Figure 3. For the excited plates, shown in Figure 4, the one subjected to 40 kHz has a mode shape where the middle of the plate has a minimum displacement amplitude compared with the other frequencies. The maximum displacement is associated with the plated subjected to 20 kHz excitation.

### 2.2. Coupling of the Plate Vibration and the Fusion Area Pressure

To identify the pressure in the fusion zone, it is assumed that the vibration is applied to a semicylinder. The relation between internal pressure coefficient $P_{f}$ and the circumferential displacement of a cylinder W can be expressed by [39]:

(6) $$P_{f}=\frac{-2B_{s}}{1-{\left(\alpha_{s}-\alpha_{f}\right)}^{2}}\frac{W}{a},$$

where $B_{s}$ and a is the bulk modulus of the semifluid and the cylinder radius, respectively. The $\alpha_{s}=k_{s}a$ and $\alpha_{f}=k_{f}a$, where $k_{s}$ and $k_{f}$ are the axial wave number of the semifluid dominated wave and the semifluid wavenumber respectively. The pressure within the semifluid can be described by a Bessel function of order zero:

(7) $$p=P_{f}J_{0}\left(k_{s}^{r}a\right),$$

where the $k_{s}^{r}$ parameter is the radial wavenumber and is related to the semifluid wavenumber $k_{f}$ by: ${\left(k_{s}^{r}\right)}^{2}=k_{f}^{2}-k_{s}^{2}$. 

**Figure 5:**
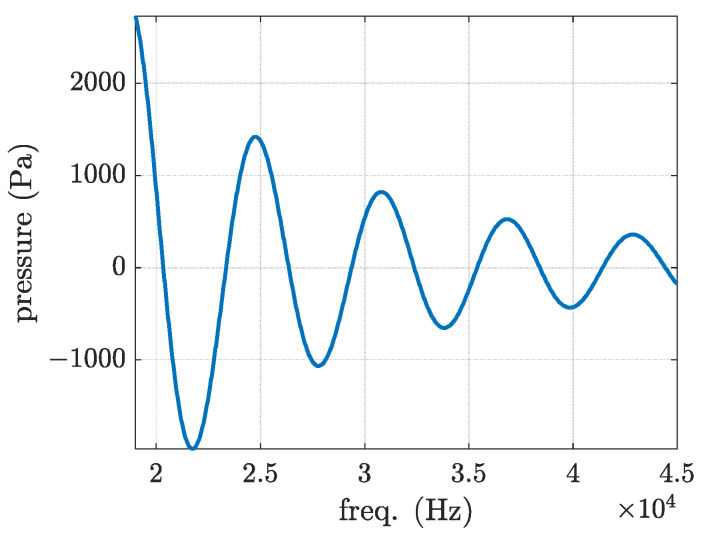
Pressure variation in the molten pool with respect to frequency.

The pressure variation in the molten pool is plotted in Figure 5 using the displacement value associated with the centre of the plate shown in Figure 4, assumed $10^{-11}$ (m). As illustrated in Figure 5, the optimum frequencies for exciting the plate are approximately 20 and 28 kHz. Considering the results shown in Figure 4 and Figure 5, it is recommended to use either a 20 or 28 kHz transducer.

### 2.3. Cavitation Dynamics

Cavitation is referred to the generation of cavities and the subsequent oscillation behaviour due to exceeding the binding force between the melt molecules [40]. The cavitation threshold in the melt is given by [41]:

(8) $$P_{B}=P_{0}-P_{v}+\frac{2}{3\sqrt{3}}{\left[\frac{{\left(\frac{2\sigma }{R_{0}}\right)}^{3}}{P_{0}-P_{v}+\frac{2\sigma }{R_{0}}}\right]}^{\frac{1}{2}},$$

where $P_{0}$ is the liquid static pressure, *P_v_* is the vapour pressure in the cavitation and σ is the surface tension coefficient of the melt. The $R_{0}$ parameter is the initial radius of the cavitation.

The relationships between the initial radius of cavitation and the sound field frequency $f_{r}$ can be expressed by [42]:

(9) $$f_{r}=\frac{1}{2{\pi R}_{0}}{\left[\frac{3\gamma }{\rho_{f}}\left(P_{0}+\frac{2\sigma }{R_{0}}\right)-\frac{2\sigma }{\rho_{f}R_{0}}\right]}^{\frac{1}{2}},$$

where γ is the specific heat capacity of the fluid and $\rho_{f}$ is the fluid density.

**Figure 6:**
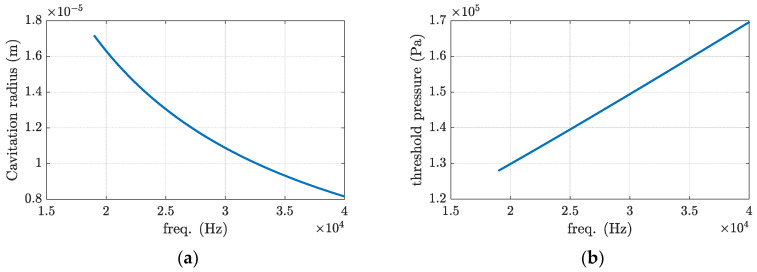
Changes to the cavitation (**a**) radius and (**b**) threshold pressure with respect to the excitation frequency.

By assuming the physical parameters *P_v_* = 2000 Pa, *σ* = 0.910 N/m, $P_{0}$ = 0.101 MPa, *R*_0_ = 10 μm, and actual cavitation threshold $P_{B}$ = 155,411 Pa, Equations (8) and (9) are plotted in Figure 6. As seen in Figure 6, increasing the frequency gives a smaller cavity but requires a higher pressure to initiate it. The required pressure to initiate cavitation is 100 times higher than the maximum value illustrated in Figure 5. Hence, using a mobile transcoder close to the area where the welding operation is happening is recommended to achieve the desired displacement level, as shown in Figure 2c.

The behaviour of cavities has been extensively studied and is covered in several literature references [40,42,43]. In this study, the oscillation of a vapour-gas cavity in an incompressible liquid is governed by the Nolting–Neppiras equation:

(10) $$R\ddot{R}+\frac{3}{2}{\dot{R}}^{2}+4\mu \frac{\dot{R}}{R}+\frac{2\sigma }{R}={(p}_{0}-P_{v}+\frac{2\sigma }{R_{0}}){\left(\frac{R_{0}}{R}\right)}^{3}-p_{0}+p_{v}+p_{A}\sin\left(\omega t\right)$$

where R, $\dot{R}$, $\ddot{R}$ are cavitation radius and its first and second derivative. The $R_{0},\;\sigma$ and μ terms are the initial radius of the cavity, the surface tension of the melt and the viscosity of the melt. The $p_{0},\;p_{v}$ and $p_{A}$ parameters are ambient pressure, sound pressure and vapour pressure, respectively.

The pressure relapsed by imploding the cavitation is proportional to the acoustic pressure of the melt. The higher the acoustic pressure of the melt, the higher the acoustic wave released by the cavitation. The critical radius can be estimated by:

(11) $$Pg+P_{v}-P_{0}=\frac{2\sigma }{R},$$

where $Pg+P_{v}$ is the total pressure inside the bubble.

The pressure distribution around the ultrasound cavitation was studied by [43], and the results indicated that the pressure reaches its maximum value at about 1.58 R from the cavitation bubble. It is shown in [44] that the weld’s penetration and width increased when pulsed ultrasound was employed.

**Figure 7:**
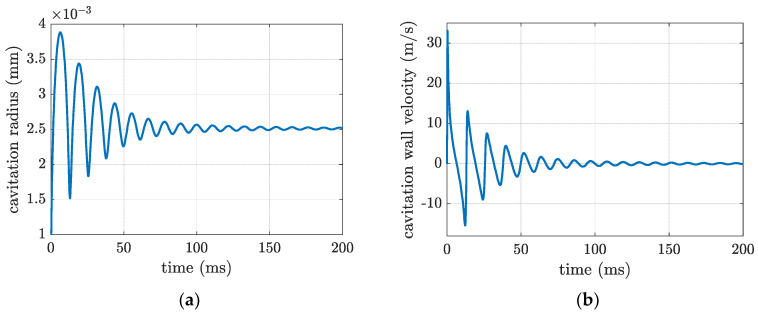
Cavitation (**a**) radius and (**b**) wall velocity subjected to 20 kHz vibration.

Changes to the cavitation radius and the wall velocity are plotted in Figure 7, using Equation (10). As seen from the plots, the ultrasound cavitation oscillation happens up to approximately 100 milliseconds. The cavitation radius and the wall velocity can reach approximately 0.004 mm and 30 m/s, respectively. The pressure distribution around the cavitation bubble was studied in [43], and the results indicated that the pressure reaches its maximum value at about 1.58 R from the cavitation bubble. Based on the results shown in Figure 7a, the effect of the shock wave from each ultrasound cavitation would be approximately $6.3\times 10^{-3}$ mm in the molten pool area. Depending on the width and the depth of the fusion zone, different cavitation numbers should be initiated to have ultrasonic grain refining.

## 3. Conclusions

This work aimed to investigate using remote-power ultrasonic vibration to improve the laser welding process. Therefore, an efficient modelling approach combining the plate vibration method and the cavitation oscillation method was employed to analyse the forced vibration of cavitation in the welding pool. The proposed method provided an accurate tool for selecting the excitation position on the plate and the frequency range required to generate cavitation in the molten pool.

Time and frequency domain studies showed that for respective frequencies of 22, 32 and 40 kHz, the displacement reached at least a level of 0.1 pm, which can excite the semifluid in the fusion zone and burst the microbubbles.

The duration of the solidifying phase should be around 0.1 s, which corresponds to 200 cycles of ultrasound at 20 kHz. Theoretically, this is sufficient time to make the bubbles expand and burst in the semifluid. Hence, grain refinement and better shaping of intermetallic can be achieved.

Future study includes manufacturing laser-welded testing specimens under ultrasonic vibration and evaluating improvements in microstructure based on welding times and ultrasound power.

## Data Availability

Not applicable.
